# Eosinophilic esophagitis: between reflux and allergy

**DOI:** 10.1186/1824-7288-41-S2-A60

**Published:** 2015-09-30

**Authors:** Alberto Ravelli

**Affiliations:** 1 Gastroenterology and GI Endoscopy Unit, University Department of Pediatrics, Children's Hospital, Brescia, Italy

## 

Eosinophilic esophagitis (EoE) is a chronic/recurrent inflammatory disease of the esophagus characterized by the association between symptoms of esophageal dysfunction and a prominent eosinophilic infiltrate (>15/HPF) of the esophageal epithelium [1]. This condition mostly affects males and is increasingly being reported in all age groups, with a prevalence currently estimated at around 1:1000 in the Western world. Many experimental and clinical observations suggest that EoE is indeed an allergic condition with a strong genetic predisposition [2]. The natural history is unknown, but several studies suggest the potential progression of untreated disease, such as escalation of symptoms from feeding difficulties in infants to strictures and food impaction in teenagers [2,3]. This evolutionary pattern is somehow similar to that of gastroesophageal reflux disease (GERD), and could be related to a histological remodeling of the esophageal wall characterized by increasing fibrosis [1,2]. Furthermore, cow's milk protein allergy often causes GERD-like symptoms, and both GERD and cow's milk allergy cause an increase eosinophil count in the esophageal mucosa of infants. Topical steroids as well as elemental or selective exclusion diet have proven effective in inducing clinical and histologic remission in EoE, but the long-term management of EoE is not yet defined [3,4]. In most pediatric studies, the only mechanism of outgrowing EoE appears to be the long-term avoidance of sensitizing food(s) rather than spontaneous remission [3]. An alternative explanation is that there are different phenotypes of EoE, including a proton pump inhibitor (PPI)-sensitive variant for which long-term PPI therapy may be indicated [5].
